# The Microbiome and Metabolome of Preterm Infant Stool Are Personalized and Not Driven by Health Outcomes, Including Necrotizing Enterocolitis and Late-Onset Sepsis

**DOI:** 10.1128/mSphere.00104-18

**Published:** 2018-06-06

**Authors:** Stephen Wandro, Stephanie Osborne, Claudia Enriquez, Christine Bixby, Antonio Arrieta, Katrine Whiteson

**Affiliations:** a Department of Molecular Biology and Biochemistry, University of California Irvine, Irvine, California, USA; b Children’s Hospital of Orange County, Orange, California, USA; Arizona State University

**Keywords:** 16S rRNA sequencing, gas chromatography, human microbiome, metabolomics, preterm infant

## Abstract

The assembly and development of the gut microbiome in infants have important consequences for immediate and long-term health. Preterm infants represent an abnormal case for bacterial colonization because of early exposure to bacteria and frequent use of antibiotics. To better understand the assembly of the gut microbiota in preterm infants, fecal samples were collected from 32 very low birth weight preterm infants over the first 6 weeks of life. Infant health outcomes included health, late-onset sepsis, and necrotizing enterocolitis (NEC). We characterized bacterial compositions by 16S rRNA gene sequencing and metabolomes by untargeted gas chromatography-mass spectrometry. Preterm infant fecal samples lacked beneficial *Bifidobacterium* spp. and were dominated by *Enterobacteriaceae*, *Enterococcus*, and *Staphylococcus* organisms due to nearly uniform antibiotic administration. Most of the variance between the microbial community compositions could be attributed to the baby from which the sample derived (permutational multivariate analysis of variance [PERMANOVA] *R*^2^ = 0.48, *P* < 0.001), while clinical status (health, NEC, or late-onset sepsis) and overlapping times in the neonatal intensive care unit (NICU) did not explain a significant amount of variation in bacterial composition. Fecal metabolomes were also found to be unique to the individual (PERMANOVA *R*^2^ = 0.43, *P* < 0.001) and weakly associated with bacterial composition (Mantel statistic *r* = 0.23 ± 0.05, *P* < 0.05). No measured metabolites were found to be associated with necrotizing enterocolitis, late-onset sepsis, or a healthy outcome. Overall, preterm infant gut microbial communities were personalized and reflected antibiotic usage.

**IMPORTANCE** Preterm infants face health problems likely related to microbial exposures, including sepsis and necrotizing enterocolitis. However, the role of the gut microbiome in preterm infant health is poorly understood. Microbial colonization differs from that of healthy term babies because it occurs in the NICU and is often perturbed by antibiotics. We measured bacterial compositions and metabolomic profiles of 77 fecal samples from 32 preterm infants to investigate the differences between microbiomes in health and disease. Rather than finding microbial signatures of disease, we found that both the preterm infant microbiome and the metabolome were personalized and that the preterm infant gut microbiome is enriched in microbes that commonly dominate in the presence of antibiotics. These results contribute to the growing knowledge of the preterm infant microbiome and emphasize that a personalized view will be important to disentangle the health consequences of the preterm infant microbiome.

## Abstract (precis)

Preterm infants face health problems likely related to microbial exposures, including sepsis and necrotizing enterocolitis. However, the role of the gut microbiome in preterm infant health is poorly understood. Microbial colonization differs from that of healthy term babies because it occurs in the NICU and is often perturbed by antibiotics. We measured bacterial compositions and metabolomic profiles of 77 fecal samples from 32 preterm infants to investigate the differences between microbiomes in health and disease. Rather than finding microbial signatures of disease, we found that both the preterm infant microbiome and the metabolome were personalized and that the preterm infant gut microbiome is enriched in microbes that commonly dominate in the presence of antibiotics. These results contribute to the growing knowledge of the preterm infant microbiome and emphasize that a personalized view will be important to disentangle the health consequences of the preterm infant microbiome.

## INTRODUCTION

Early-life exposure to microbes and their metabolic products is a normal part of development, with an enormous and underexplored impact on the immune system. The intestinal microbiota of infants initially assembles by exposure to the mother’s microbiota and microbes in the environment (1–4). In healthy breastfed infants, Bifidobacterium longum sp. infantis strains capable of digesting human-milk oligosaccharides dominate the infant gut (5). When infants are born preterm, they are exposed to environmental and human-associated microbes earlier in their development than normal and rarely harbor *Bifidobacterium* spp. in their gut communities. We do not yet understand the effects of altering the timing of initial bacterial exposure. With numerous emerging health consequences related to the microbiome, understanding factors that influence this initial assembly of the microbiome will be important.

Preterm infants are routinely treated with antibiotics, which enriches for microbes that can colonize in the presence of antibiotics (4, 6, 7). While antibiotics have tremendously reduced infant mortality, their effect on microbiota assembly and resulting health consequences is not fully understood. Prenatal and postnatal antibiotics have been shown to reduce the diversity of the infant intestinal microbiota (8, 9). Children under 2 years of age are prescribed antibiotics at a higher rate than any other age group, and 85% of extremely low birth weight infants (<1,000 g) are given at least one course of antibiotics (10). Even if an infant is not exposed to antibiotics after birth, approximately 37% of pregnant women use antibiotics over the course of the pregnancy (11).

Perturbing the microbiota of infants can have immediate and long-lasting health consequences. In the short term, infants can be infected by pathogenic bacteria that result in sepsis, which is categorized as early onset or late onset, depending on the timing after birth. Preterm infants are also at high risk of developing necrotizing enterocolitis (NEC), which is a devastating disease that causes portions of the bowel to undergo necrosis. NEC is one of the leading causes of mortality in preterm infants, who make up 90% of NEC cases (12). The incidence of NEC among low birth weight preterm infants is approximately 7% and causes death in about one-third of cases. The exact causes of NEC are not known, but an excessive inflammatory response to intestinal bacteria may be involved (13).

Many of the long-term consequences of microbial colonization are believed to be mediated by interactions between the intestinal microbiota and the immune system. In addition to interacting directly with the immune system, the microbiota interacts with the immune system through the production of metabolites that can be taken up directly by immune and epithelial cells (14, 15). For example, bacterial production of short-chain fatty acids can affect the health and integrity of the intestinal epithelia and immune cells (16–18). However, few studies use metabolites alongside bacterial community profiling. In fact, the healthy composition of an infant fecal metabolome is understudied.

In this retrospective study, we follow changes in the gut microbiota over time in 32 very low birth weight (<1,500-g) preterm infants born in Children’s Hospital, Orange County, Orange, CA. We simultaneously track their bacterial compositions and metabolite profiles over time. Infants were classified into three groups based on health outcomes: healthy, late-onset sepsis, and NEC. The composition of the intestinal microbiota was measured by 16S rRNA gene sequencing of fecal samples taken over time. Preterm infant guts were dominated by *Enterobacteriaceae*, *Enterococcus*, and *Staphylococcus* organisms. Untargeted metabolomics analysis of the fecal samples by gas chromatography mass spectrometry (GC-MS) revealed a personalized metabolome that was weakly associated with the bacterial composition.

## RESULTS

### Patient cohort.

**FIG 1:**
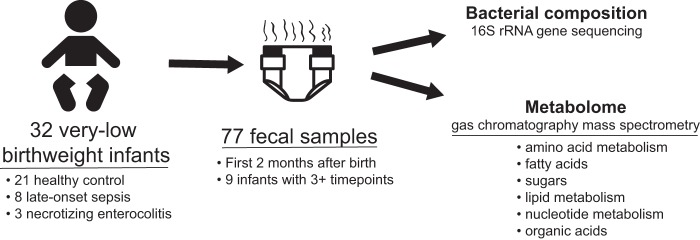
Study design schematic. Longitudinal fecal samples were collected over the first 75 days of life from very low birth weight infants in the NICU. Bacterial compositions and metabolomes were characterized.

A total of 77 fecal samples were collected from 32 very low birth weight infants in the neonatal intensive care unit (NICU) at Children’s Hospital, Orange County, Orange, CA, from 2011 to 2014 (Table 1; Fig. 1). Birth weights ranged from 620 to 1,570 g. Fecal samples were collected between days 7 and 75 of life. Sampling times and numbers of fecal samples varied. Three or more longitudinal samples were available from 10 of the infants, while one or two samples were available from the remaining 22 infants. Three infants developed NEC, 8 developed late-onset sepsis, and 21 remained healthy. Twelve infants were delivered vaginally, while the remaining 22 were delivered by caesarean section. All infants were fed either breastmilk or a combination of breastmilk and formula. Twenty-four infants had a record of receiving antibiotics at some point during the sampling period, the most common being ampicillin and gentamicin.

**TABLE 1:** Clinical and sampling information for all infants^a^

Infant	No. of samples	Age(s) at which sample(s) was collected (days)	Age at disease onset (days)	Group	Age at birth (wks, days)	Birth wt (g)	Antibiotics administered	Delivery mode	Food	Twin set
1	2	7, 7		Control	27, 4	875		CS	BM	
2	3	15, 15, 36		Control	31	1,570	AMP, GEN	CS	BM, F	
3	1	19		Control	26	980	AMP, GEN	CS	BM	
4	2	11, 11		Control	30, 3	1,335		V	BM	
5	2	18, 18		Control	24, 5	630		CS	BM	
6	4	25, 26, 28, 43		Control	28, 5	860	AMP, GEN	CS	BM, F	
7	3	10, 21, 24		Control	25, 2	885		CS	BM, F	
8	1	10		Control	25, 4	940	AMP, GEN	V	BM	
9	1	8		Control	27, 2	1,205		V	BM	
11	2	29, 29		Control	27, 4	850	AMP, GEN	V	BM	
12	1	22		Control	26, 2	880	AMP, GEN	CS	BM, F	1
13	1	23		Control	26, 2	925	AMP, GEN	CS	BM, F	1
14	1	8		Control	31, 4	1,190	AMP, GEN	CS	BM	
15	3	18, 40, 40		Control	28, 1	1,270	AMP, GEN	CS	BM, F	2
16	1	19		Control	28, 1	1,355	AMP, GEN	CS	BM, F	2
17	3	18, 32, 54		Control	26, 2	660	AMP, GEN	CS	BM	
21	1	10		Control	28, 6	1,180	AMP, GEN	CS	BM	
22	1	25		Control	28, 6	1,360	AMP, GEN	V	BM, F	
24	2	27, 73		Control	26	740	AMP, GEN	CS	BM	3
25	1	28		Control	26	780	AMP, GEN	CS	BM	3
35	2	18, 18		Control	25, 5	920		CS	BM, F	
23	7	14, 15, 27, 28, 30, 30, 56	27	NEC	26, 6	1,080	AMP, GEN, CTX, VAN	V	BM	
28	4	31, 32, 33, 48	31	NEC	26	1,060	VAN, PIP	CS	BM, F	
30	4	21, 41, 42, 56	41	NEC	23, 6	620	CFZ, AZM, AMP	V	BM, F	
20	1	21	26	Septic	24, 5	815	AMP, GEN	CS	BM	
10	6	15, 35, 36, 37, 39, 40	27	Septic	26, 5	940	AMP, GEN, VAN	V	BM, F	
26	1	22	22	Septic	24, 4	660	AMP, GEN, CTX, VAN	CS	BM	4
27	2	22, 31	29	Septic	24, 5	650	AMP, GEN	CS	BM	4
29	2	20, 26	26	Septic	26, 1	980	CTX, VAN	CS	BM	
31	5	10, 34, 35, 38, 45	34	Septic	27	710	AMP, GEN	CS	BM, F	
32	4	32, 32, 53, 75	32	Septic	27, 5				BM, F	
37	3	8, 17, 18	13	Septic	24, 1	680	AMP, GEN, CFZ, OXA	V	BM	

a AMP, ampicillin; CTX, cefotaxime; CFZ, cefazolin; GEN, gentamicin; OXA, oxacillin; PIP, piperacillin; VAN, vancomycin; CS, C-section; V, vaginal; BM, breast milk; F, formula.

### Microbial community characterization.

**FIG 2:**
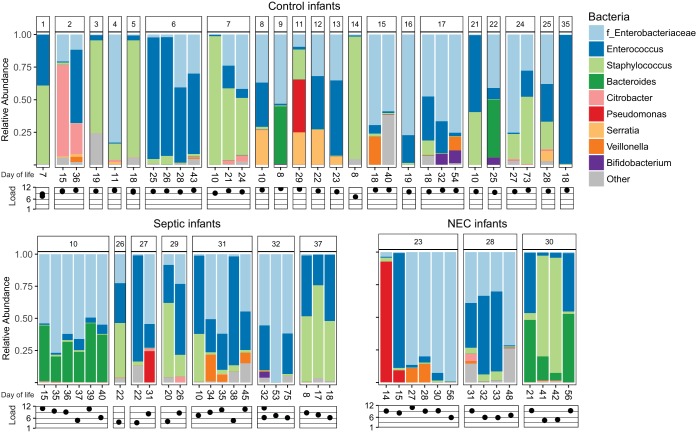
Bacterial composition and bacterial load of preterm infant guts. Stacked barplots show relative abundances of bacteria at the genus level in all infant samples. The family (f) *Enterobacteriaceae* is included because genus-level resolution was not available. A log-scale relative bacterial load is shown underneath each sample.

We sequenced the 16S rRNA gene content of each fecal sample to determine bacterial composition. The total bacterial load of each fecal sample was measured by quantitative PCR (qPCR) of the 16S rRNA gene and scaled to the total weight of stool from which the DNA was extracted. Among all infants, bacterial abundances varied over 4 orders of magnitude and were lower in infants that developed NEC or late-onset sepsis (*P* < 0.001) (Fig. 2). The high variation in bacterial load is likely due to the nearly uniform use of antibiotics. Bacterial communities were composed of mostly* Enterobacteriaceae*, *Enterococcus*, *Staphylococcus*, and *Bacteroides* organisms (Fig. 2). Most samples were dominated by one to three genera of bacteria. Only three infants (two fed breastmilk, one fed breastmilk and formula) were colonized at a >1% relative abundance by bifidobacteria, which emerging evidence suggests are key members of the infant microbiome. However, we note that the primers used are able to detect 30% (1,741 out of 5,146) of the bifidobacterial species represented in the Ribosome Project Database, including 38 *Bifidobacterium infantis* substrains, versus 68% (2,177,663 out of 3,196,041) of all bacterial species in the database (19). No single bacterial operational taxonomic unit (OTU) or community composition was consistently found for infants that became sick (NEC or late-onset sepsis) compared to that of infants that remained healthy.

**FIG 3:**
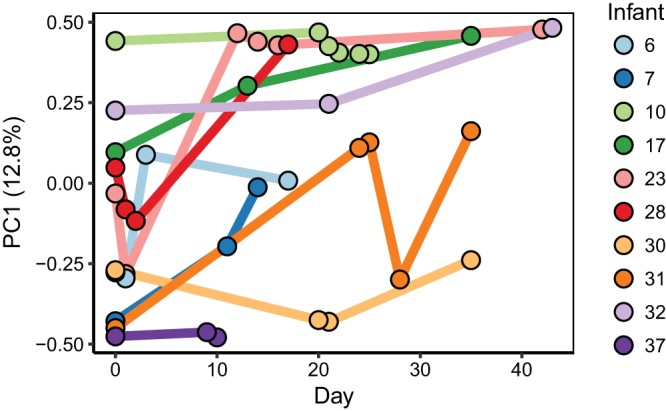
First axis of PCoA based on weighted UniFrac distances between bacterial communities plotted over time. Each dot represents a single fecal sample and is colored by infant. Lines connect samples for each infant to show change over time. The results shown are only for infants with three or more longitudinal samples. PC1, principal component 1.

Longitudinal sampling revealed that over the course of days, the bacterial composition could change dramatically (Fig. 3). Permutational multivariate analysis of variance (PERMANOVA) was applied to determine which of the known clinical factors explained the most variance in the bacterial community compositions. The individual explained 48% (*P* < 0.001) of the variance in the samples, meaning that about half of the total variance among all tested fecal samples could be attributed to the infant from which the fecal sample came (see Table S1a in the supplemental material). Delivery mode explained a smaller proportion of variance (12% variance, *P* < 0.05), but none of the other factors explained a significant amount of variation in the bacterial compositions, including infant health, overlapping dates in the NICU, or feeding mode. Only vaginally born infants were colonized by *Bacteroides* (four out of nine infants), while none of the 22 infants born by C-section were colonized. Eight of the infants in the study are twins. Twin set 1 (infants 12 and 13) had similar microbial compositions, while the other three sets of twins did not (Fig. S1).

10.1128/mSphere.00104-18.1 FIG S1  Bacterial composition of each set of twins. Download FIG S1, EPS file, 2 MB. Copyright © 2018 Wandro et al. 2018 Wandro et al. This content is distributed under the terms of the Creative Commons Attribution 4.0 International license.

10.1128/mSphere.00104-18.3 TABLE S1  PERMANOVA for clinical factors explaining the differences in fecal bacterial composition (a) and metabolome (b). Bold factors are statistically significant. PERMANOVA for health outcome tests for differences among control, NEC, and late-onset sepsis groups. PERMANOVA results for health and individual include only infants with three or more longitudinal samples. Download TABLE S1, DOCX file, 0.01 MB. Copyright © 2018 Wandro et al. 2018 Wandro et al. This content is distributed under the terms of the Creative Commons Attribution 4.0 International license.

**FIG 4:**
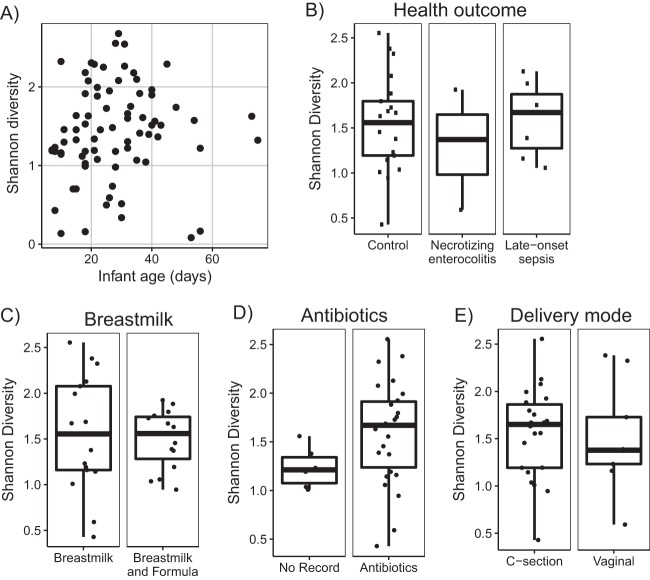
Alpha diversity as measured by the Shannon index of bacterial composition. (A) Alpha diversities of all samples over the age of the infant. Boxplots of the average alpha diversity of each infant are separated by health outcome (B), infants that were fed only breastmilk or a combination of formula and breastmilk (C), record of antibiotic usage (D), and delivery mode (E).

The diversity of the bacterial communities was low, as expected for preterm infants. Alpha diversity as measured by the Shannon index increased overall with age, but the trend was not significant (linear model *R*^2^ = 0.005, *P* = 0.52) (Fig. 4A). Other clinical factors, including health outcome, feeding (breastmilk versus breastmilk and formula), antibiotic use, and delivery mode were tested for an effect on the alpha diversity (Fig. 4B to E). None of the factors were associated with a difference in alpha diversity except recorded antibiotic use, in which Shannon diversity was unexpectedly lower on average in infants that did not have a record of receiving antibiotics (Wilcoxon rank sum test *P* = 0.06). It should be noted that although six infants did not have a record of antibiotic use, records may be incomplete due to hospital transfers.

### Metabolomics.

**FIG 5:**
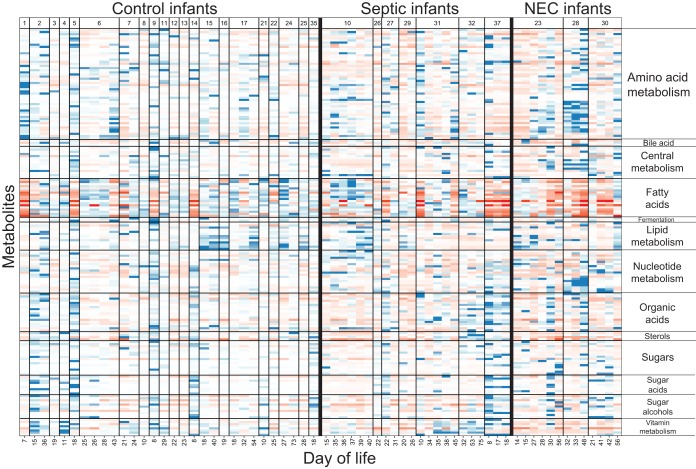
Metabolite profile of preterm infant fecal samples. Color indicates the modified *z* score, which is based on the median intensity for each metabolite in all infant samples. Red cells indicate standard deviations below the median, and blue cells indicate standard deviations above the median value for each metabolite. Measured metabolites that could be assigned to a category are shown. Samples on the *x* axis are grouped by infant and ordered longitudinally. Metabolites within each category are listed in the supplemental material.

**FIG 6:**
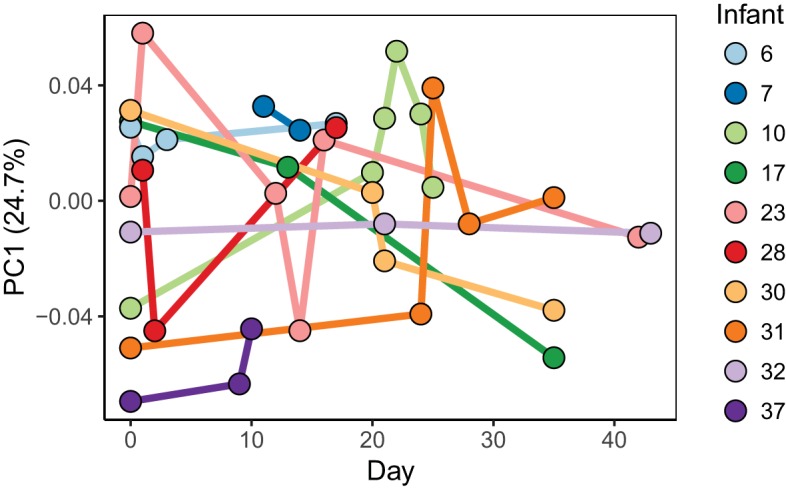
First component of PCoA of the metabolite profile over time. Manhattan distances between samples were calculated and visualized by PCoA. The first principal component which explains the most variation among the samples is shown over time. Each dot represents a single fecal sample and is colored by infant. Lines connect samples for each infant to show change over time. The results shown are only for infants with three or more longitudinal samples.

Metabolite profiles of infant fecal samples were analyzed by gas chromatography-mass spectrometry, which measures small primary metabolites. Over 400 small molecules were detected from each fecal sample, and 224 metabolites were known compounds. Metabolites were grouped into the following categories: amino acid metabolism, bile acids, central metabolism, fatty acids, fermentation products, lipid metabolism, nucleotide metabolism, organic acids, sterols, sugars, sugar acids, sugar alcohols, and vitamin metabolism (Fig. 5; Table S2). No metabolites or categories of metabolites were found to be associated with necrotizing enterocolitis or late-onset sepsis. The metabolite profile of each infant was seen to vary over time, and the variation was similar to that seen in the bacterial composition (Fig. 6). PERMANOVA to determine which factors explain the most variance in the metabolite profile indicate that the individual explains 43% (*P* < 0.001) of the variation (Table S1b).

10.1128/mSphere.00104-18.4 TABLE S2  All identified metabolites measured by GC-MS and their assigned categories. Download TABLE S2, PDF file, 0.1 MB. Copyright © 2018 Wandro et al. 2018 Wandro et al. This content is distributed under the terms of the Creative Commons Attribution 4.0 International license.

To determine which metabolites might be useful for tracking bacterial metabolism in the infant gut, we examined metabolites with consistent abundances among infants versus those that varied (Fig. S2). In general, sugars, central metabolites, and amino acids varied, while fatty acids, sterols, organic acids, and bile acids were more consistent. Infant 23, who developed necrotizing enterocolitis at day 16 of life, had low abundances of amino acid metabolites the 2 days prior to disease onset (Fig. 5). However, several of the healthy control infants also had similarly low abundances of amino acid metabolites. The individual signal of each infant’s metabolome is far more evident than any trends due to clinical factors (Table S1b).

10.1128/mSphere.00104-18.2 FIG S2  Average variation among infants of each metabolite grouped by category. Each dot represents a single metabolite. The coefficient of variation for each metabolite was calculated as the standard deviation divided by the mean intensity of that metabolite in all samples from all infants. Download FIG S2, EPS file, 1.8 MB. Copyright © 2018 Wandro et al. 2018 Wandro et al. This content is distributed under the terms of the Creative Commons Attribution 4.0 International license.

### Bacterial composition associated with metabolite profile.

Bacterial metabolism in the gut is expected to contribute to the abundances of metabolites detected in fecal samples. We wanted to know whether fecal samples with similar bacterial compositions were also similar in their metabolite profiles. We employed a Mantel test using Pearson correlations between distances among bacterial compositions of samples and distances among metabolite profiles of samples. Because bacterial compositions and metabolite profiles are personalized, using multiple samples from a single infant would skew the result. Therefore, one sample from each infant was randomly selected 100 times to remove the effect of the individual, and the Mantel test was applied to each subset. The average Mantel statistic (*r* = 0.23 ± 0.05, *P* < 0.05) indicates a weak but significant association between the bacterial composition and metabolite profile. Also, within individual infants, shifts in the bacterial composition are accompanied by shifts in the metabolome. Infants 17, 23, and 31 had dramatic shifts in both bacterial composition and metabolome profile over time, while infants 10 and 37 remained stable in both their bacterial composition and their metabolome.

**FIG 7:**
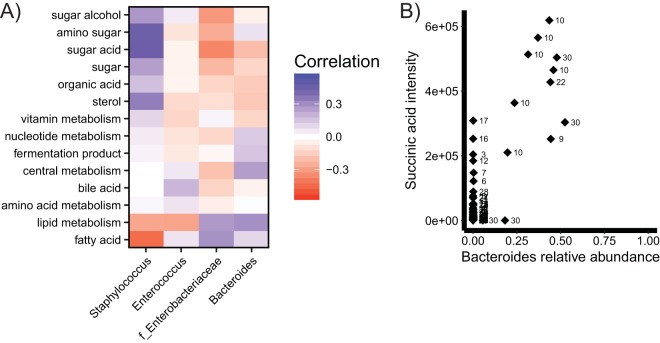
Correlations between bacterial abundances and metabolite intensities. (A) Averages of correlations between bacterial abundances and all metabolites in each metabolite category; (B) correlations between *Bacteroides* abundance and succinic acid intensity in all samples. Numbers indicate infant numbers.

To investigate the correlations driving this overall association, we calculated correlations between bacterial abundances and metabolite intensities (Fig. 7A). *Staphylococcus* had the most positive correlations, including several classes of sugar metabolites, organic acids, and central metabolites. Fatty acids, lipid metabolism, and amino acids were positively correlated with the commonly abundant gut colonizers *Enterobacteriaceae* and *Bacteroides* and negatively correlated with the common low-abundance colonizers *Staphylococcus* and *Enterococcus*. We also looked more specifically at individual metabolites correlated with bacterial abundances (Fig. 7B). Bacteroidetes were found to be positively correlated with succinate (*r* = 0.85). Many other weak correlations (*r* < 0.5) exist between bacterial abundances and metabolite intensities, but the sample size is not large enough to distinguish signal from noise.

## DISCUSSION

Bacterial compositions in this cohort were consistent with the emerging picture from other studies showing that the preterm infant gut harbors communities dominated by facultative anaerobes, including *Enterobacteriaceae*, *Enterococcus*, and *Staphylococcus* (1, 2, 20). These communities appear to be enriched in commonly antibiotic-resistant organisms (21). While we expected to find associations between bacterial community composition and health outcome in this cohort, we were surprised to find that there were not clear signatures of microbiome composition linked to NEC or sepsis. In larger cohorts, associations between particular bacteria or metabolites with NEC have been reported; however, they are not universal signatures across patients and may reflect patient variation more than disease etiology (22–25). In fact, the strongest signal in both the microbiome and metabolome data from this cohort was the infant from whom the sample was taken. Overall, preterm infant microbiomes in this study were shaped by antibiotics, which have a strong impact on all patients, regardless of health outcome.

Although the bacterial composition of infant guts varied over time, we saw that longitudinal samples from individual infants remained highly personalized over several weeks; nearly half of the variation in the microbial community compositions can be explained by the individual from which the sample came. The stability of animal-associated microbiomes is an active area of research, with studies finding that the individual microbiome of an adult remains stable through time (26) but can be perturbed by extreme changes in diet or antibiotics (27–29). The bacterial composition in the adult gut largely returns to its previous state 1 month after antibiotic treatment, but altering the initial assembly of the microbiota in infants can have long-lasting health consequences (7, 27, 30, 31). Previous work has found ampicillin and gentamicin (the most common antibiotics taken by infants in this study) to have an inconsistent effect on bacterial diversity, sometimes increasing and sometimes decreasing diversity (1). Similarly, in these infants, ampicillin and gentamicin resulted in more variation in bacteria, but there was no clear trend of increasing or decreasing diversity. However, antibiotics change the dominant members of the microbiota, which might have profound effects on immune development and growth (7, 31–33).

Evidence that a healthy infant gut microbiota is dominated by bifidobacteria with the capacity to digest human milk oligosaccharides in breastmilk is emerging (5, 34, 35). The lack of a core bifidobacterial community in infants might leave the microbiota open to colonization by facultative anaerobes, as we observed in these infants (36). Proteobacteria, such as *Enterobacteriaceae*, are commonly seen to increase in abundance after antibiotic administration (21, 37, 38). Indeed, infants in this study had microbiomes that were shaped by antibiotic use. Although 6 of the 32 infants in this study did not have recorded antibiotic use around sampling time, their microbiotas could still have been affected by prenatal antibiotics taken by the mother (7, 31, 39).

Microbiome studies have become widespread, so that a typical bacterial composition is well characterized in a range of sample cohorts. However, the same cannot be said for the metabolome. There is a dearth of knowledge about what a consensus healthy infant fecal metabolome should be, making comparisons for the cohort in this study difficult. To add to the complexity, each metabolomic approach detects subsets of metabolites and depends on sample extraction and other method choices. Increasing the frequency of metabolomic data collection in microbiome studies would be a huge step forward for the field. Baseline knowledge about the typical connections between metabolite abundances and bacterial metabolism should be collected so that molecules that have consistent abundances in a healthy state can give context to data generated from clinical samples in different disease states.

Untargeted metabolomics can survey many metabolites in a biological sample to provide a snapshot of the active metabolism in a system such as the human gut. Metabolite profiles of preterm infants in this study were found to be personalized to a degree similar to that of the bacterial composition. This is in contrast to results of a previous study on full-term infants that showed the metabolomic profile to be stable and weakly associated with bacterial composition over the first few years of life (40). Personalized metabolic signatures of disease hold great promise to complement microbiota profiling in human systems (18, 36). However, analyzing metabolomic data is challenging because an array of inputs contribute to the abundances of metabolites in fecal samples, including bacterial metabolism, host biology, and food intake.

We report a number of correlations between bacteria and metabolites in preterm infant feces, and bacterial metabolism has previously been shown to contribute to metabolite abundances in humans and mice (14, 15, 41). Short-chain fatty acids are now commonly measured and associated with bacterial fermentation in the gut (42). In this study, the only short-chain fatty acid detected was succinate, which we found to be correlated with the presence of *Bacteroides*, which produces acetate and succinate from carbohydrate fermentation (43). We also detected several medium-chain fatty acids, which were generally correlated with the abundance of *Bacteroides* and *Enterobacteriaceae*. None of the 22 C-section-born infants in this study were colonized by *Bacteroides*, possibly due to a lack of vertical transmission from the mother during birth (3).

Overall, we find that preterm infant microbiomes are shaped by shared exposures, especially to antibiotics, leading to the dominance of antibiotic-resistant facultative anaerobes, such as *Enterococcus* spp. The anaerobic, milk-degrading bifidobacteria were largely absent, even in preterm infants with access to breastmilk, possibly due to a lack of exposure to microbes from family members in the sterile hospital environment, along with antibiotics. Our understanding of the health consequences of microbial colonization under these antibiotic-enriched circumstances is still in its infancy.

## MATERIALS AND METHODS

### Sample collection.

Stool samples from diapers of preterm infants in the neonatal intensive care unit at Children’s Hospital, Orange County, CA, were collected by nurses over 3 years from 2011 to 2014. Samples were immediately stored at −20°C and then transferred to −80°C no more than 3 days postcollection. Samples were kept at −80°C and thawed once for DNA extraction and metabolomics. A total of 77 stool samples were collected from 32 preterm infants.

### DNA extraction and 16S rRNA gene sequencing.

Stool samples were thawed once, and DNA was extracted from ~50 mg using a Zymo fecal DNA miniprep kit (D6010). The V3 and V4 regions of the 16S rRNA gene were amplified by a two-stage PCR. The first PCR amplified the V3-to-V4 region of the 16S rRNA gene using the 341F and 805R primers: 5′-CCTACGGGNGGCWGCAG-3′ (forward primer) and 5′-GACTACHVGGGTATCTAATCC-3′ (reverse primer) (44). These primers also added an overhang so that barcodes and Illumina adaptors could be added in the second PCR. The first PCR was done as follows: 30 cycles of 95°C for 30 s, 65°C for 40 s, and 72°C for 1 min. Immediately after completion of the first PCR, primers with sample-specific barcodes and Illumina adaptor sequences were added and a second PCR was performed as follows: 9 cycles of 94°C for 30 s, 55°C for 40 s, and 72°C for 1 min. PCRs were cleaned using Agencourt AMPure XP magnetic beads (A63880) by the recommended protocol. Amplicons were run on an agarose gel to confirm amplification and then pooled. The amplicon pool was run on an agarose gel, and the 500-bp fragment was cut out and gel extracted using a Millipore gel extraction kit (LSKGEL050). The sequencing library was quantified using Quant-iT Pico Green double-stranded DNA (dsDNA) reagent and sent to Laragen Inc. for sequencing on the Illumina MiSeq platform with 250-bp paired-end reads, producing a total of 2.4 million paired-end reads.

### qPCR for bacterial load.

The bacterial load of each fecal sample was measured with quantitative PCR (qPCR) for a conserved region of the 16S rRNA gene. The following primers were used: 5′-TCCTACGGGAGGCAGCAGT-3′ and 5′-GGACTACCAGGGTATCTAATCCTGTT-3′. PerfeCTa SYBER Green SuperMix reaction mix (Quantabio; 95054) was used to quantify DNA from samples. Abundances of 16S rRNA genes relative to the mass of stool were determined for each sample. Total fecal DNA was measured with a Quant-iT Pico Green dsDNA assay kit (ThermoFisher; P11496).

### Sequence processing.

Sequences were quality filtered using PRINSEQ to remove adaptors as well as any sequences that were less than 120 bp, contained any ambiguous bases, or had a mean Phred quality score of less than 30 (45). Reads were found to drop steeply in quality after 140 bp, so all reads were trimmed to 140 bp. The forward read contained the V3 region in the high-quality first 140 bp, while the V4 region was captured in the low-quality region of the reverse reads. Therefore, we used only the forward reads for subsequent analyses.

### Bacterial community analysis.

Quantitative Insights into Microbial Ecology (QIIME) was used for *de novo* OTU picking with the Swarm algorithm, with a clustering threshold of 8 (46, 47). This resulted in 2,810 OTUs among all samples. OTUs containing only one sequence were filtered out, leaving 212 OTUs. Taxonomy was assigned to each OTU using QIIME and the Greengenes 13_8 database. An OTU table was constructed and used for downstream analysis. Ten rarefactions were performed on the OTU table down to 2,000 reads per sample, which was the largest number of reads that allowed retention of most samples. QIIME was used to calculate alpha diversity by the Shannon index and beta diversity by the average weighted UniFrac distance of the 10 rarefactions. Community composition barplots, principal-coordinate analysis (PCoA) plots, and alpha diversity plots were created using R and the ggplot2 package (48, 49). All R scripts are included in the supplemental material.

### Untargeted metabolomics by GC-MS.

When fecal samples were thawed for DNA extraction, approximately 50 mg was collected and refrozen at −80°C for metabolomics. Samples were sent on dry ice to the West Coast Metabolomics Center (WCMC) at UC, Davis, for untargeted metabolomics by gas chromatography-time of flight mass spectrometry. Metabolites were extracted from fecal samples with a 3:3:2 mixture of isopropanol, acetonitrile, and water, respectively, before derivatization and GC-MS analysis by Fiehn lab standard operating procedures (50–52). Metabolite profiles were compared by calculating Manhattan distances between samples based on all metabolite intensities and visualized by PCoA using the vegan and ape packages in R (53, 54).

### PERMANOVA.

PERMANOVA was used to determine factors that explained variance in bacterial community compositions and metabolite profiles. PERMANOVA was performed using the Adonis function in the vegan package in R. The input for PERMANOVA was a UniFrac distance matrix of the 16S rRNA data and Manhattan distances of the metabolite profiles. Briefly, PERMANOVA quantifies the variation among samples explained by the given groupings compared to randomized groupings. To measure the variance explained by an individual infant, we excluded samples that had fewer than three longitudinal samples, leaving 10 infants. To measure the variance explained by health outcome, we again included only infants with three or more longitudinal samples, and groups were permuted among infants, not samples, so that the effect of the individual would be minimal. When performing PERMANOVA for factors other than individual, we accounted for the longitudinal sampling by averaging samples from each individual.

### Correlations between bacteria and metabolites.

Pearson correlations between bacterial abundances and normalized metabolite intensities were calculated using the cor function in R. Correlations were calculated between the relative abundances of all bacterial classes and all metabolite intensities among all samples from all infants. Only the four most highly abundant genera of bacteria were used to ensure that no results were skewed by taxa present in only one or a few samples. For each class of metabolite, the average of all correlations between metabolites in that class and each taxon was calculated so that trends between bacterial taxa and classes of metabolites could be visualized with a heatmap.

### Mantel test.

To determine whether fecal samples with similar bacterial compositions also had similar metabolite profiles, a Mantel test was performed. To account for the effect of longitudinal sampling, each data set was randomly subsampled down to one sample per infant. A Bray-Curtis dissimilarity matrix was computed for the bacterial composition, and Manhattan distances were calculated for metabolite intensities. The Mantel function in the vegan package of R was used to calculate the Mantel statistic for a Pearson correlation between the two dissimilarity matrices. The averages and standard deviations of the Mantel statistic *r* and the *P* value for the 100 Mantel tests were reported.

### Data availability.

Raw sequence data are available in the SRA database under accession number SRP137076. OTU tables, raw metabolomics data, a markdown file of sequence processing workflow, and R scripts used for analyses are available at https://github.com/swandro/preterm_infant_analysis.
