# Supplementary material for: The Microbiome and Metabolome of Preterm Infant Stool Are Personalized and Not Driven by Health Outcomes, Including Necrotizing Enterocolitis and Late-Onset Sepsis
Source: mSphere. 2018 Jun 6;3(3):e00104-18. doi: 10.1128/mSphere.00104-18 (PMC5990886; doi:10.1128/mSphere.00104-18)
Supplement: TABLE S1 [file sph003182562st1.docx]

a)

| (Microbiome composition) | **Df** | **Variance explained (R^2^)** | **P-value** |
| --- | --- | --- | --- |
| Health | 2 | 0.079 | 0.64 |
| **Individual** | **9** | **0.48** | **< 0.001** |
| **Delivery mode** | **1** | **0.12** | **0.046** |
| Breastmilk/Forumla | 1 | 0.07 | 0.076 |
| Antibiotics | 1 | 0.039 | 0.29 |
| Date in NICU | 6 | 0.25 | .335 |

b)

| (Metabolite composition) | **Df** | **Variance explained (R^2^)** | **P-value** |
| --- | --- | --- | --- |
| Health | 2 | 0.15 | 0.095 |
| **Individual** | **9** | **0.48** | **< 0.001** |
| Delivery mode | 1 | 0.03 | 0.55 |
| **Breastmilk/Forumla** | **1** | **0.13** | **0.005** |
| Antibiotics | 1 | 0.045 | 0.181 |
