# Supplementary material for: The Microbiome and Metabolome of Preterm Infant Stool Are Personalized and Not Driven by Health Outcomes, Including Necrotizing Enterocolitis and Late-Onset Sepsis
Source: mSphere. 2018 Jun 6;3(3):e00104-18. doi: 10.1128/mSphere.00104-18 (PMC5990886; doi:10.1128/mSphere.00104-18)
Supplement: TABLE S2 [file sph003182562st2.pdf]

Metabolite	Category
2-ketoisocaproic acid	amino acid metabolism
2-picolinic acid	amino acid metabolism
3,4-dihydroxyphenylacetic acid	amino acid metabolism
3-phenyllactic acid	amino acid metabolism
4-aminobutyric acid	amino acid metabolism
4-hydroxybenzoate	amino acid metabolism
4-hydroxyphenylacetic acid	amino acid metabolism
5-aminovaleric acid	amino acid metabolism
alanine	amino acid metabolism
alanine-alanine	amino acid metabolism
asparagine	amino acid metabolism
aspartic acid	amino acid metabolism
beta-alanine	amino acid metabolism
cysteine	amino acid metabolism
cystine	amino acid metabolism
glutamine	amino acid metabolism
glutaric acid	amino acid metabolism
glycine	amino acid metabolism
histidine	amino acid metabolism
homocystine	amino acid metabolism
homoserine	amino acid metabolism
indole-3-lactate	amino acid metabolism
isoleucine	amino acid metabolism
leucine	amino acid metabolism
lysine	amino acid metabolism
methionine	amino acid metabolism
methionine sulfoxide	amino acid metabolism
N-acetylaspartic acid	amino acid metabolism
n-acetylglutamate	amino acid metabolism
N-acetylornithine	amino acid metabolism
N-acetylputrescine	amino acid metabolism
N-methylalanine	amino acid metabolism
O-acetylserine	amino acid metabolism
ornithine	amino acid metabolism
phenol	amino acid metabolism
phenylacetic acid	amino acid metabolism
phenylalanine	amino acid metabolism
phenylethylamine	amino acid metabolism
pipecolinic acid	amino acid metabolism
proline	amino acid metabolism
putrescine	amino acid metabolism
pyrrole-2-carboxylic acid	amino acid metabolism
serine	amino acid metabolism
shikimic acid	amino acid metabolism
threonine	amino acid metabolism
trans-4-hydroxyproline	amino acid metabolism
tryptophan	amino acid metabolism
tyramine	amino acid metabolism
tyrosine	amino acid metabolism
urea	amino acid metabolism
urocanic acid	amino acid metabolism
valine	amino acid metabolism
N-acetyl-D-galactosamine	amino sugar
n-acetyl-d-hexosamine	amino sugar
chenodeoxycholic acid	bile acid
cholic acid	bile acid
taurine	bile acid
aconitic acid	central metabolism
alpha-ketoglutarate	central metabolism
citramalic acid	central metabolism
citric acid	central metabolism
dihydroxyacetone	central metabolism
fumaric acid	central metabolism
glucose-6-phosphate	central metabolism
glyceric acid	central metabolism
isocitric acid	central metabolism
malic acid	central metabolism
oxalic acid	central metabolism
pyrophosphate	central metabolism
pyruvic acid	central metabolism
succinic acid	central metabolism
sulfuric acid	central metabolism
2-hydroxybutanoic acid	fatty acid
arachidic acid	fatty acid
arachidonic acid	fatty acid
behenic acid	fatty acid
capric acid	fatty acid
cerotinic aci	fatty acid
elaidic acid	fatty acid
heptadecanoic acid	fatty acid
isoheptadecanoic acid NIST	fatty acid

lauric acid	fatty acid	xanthine	nucleotide metabolism
lignoceric acid	fatty acid	xanthosine	nucleotide metabolism
linoleic acid	fatty acid	2,3-dihydroxybutanoic acid NIST	organic acid
myristic acid	fatty acid	2-deoxytetronic acid	organic acid
nonadecanoic acid	fatty acid	2-deoxytetronic acid NIST	organic acid
oleic acid	fatty acid	2-hydroxyglutaric acid	organic acid
palmitic acid	fatty acid	2-hydroxyhexanoic acid	organic acid
pentadecanoic acid	fatty acid	2-hydroxyvaleric acid	organic acid
stearic acid	fatty acid	3-hydroxy-3-methylglutaric acid	organic acid
butane-2,3-diol NIST	fermentation product	3-hydroxybutyric acid	organic acid
lactic acid	fermentation product	adipic acid	organic acid
1-monoheptadecanoyl glyceride NIST	lipid metabolism	azelaic acid	organic acid
1-monoolein	lipid metabolism	benzoic acid	organic acid
1-monopalmitin	lipid metabolism	digalacturonic acid	organic acid
1-monostearin	lipid metabolism	erythronic acid lactone	organic acid
2-monoolein	lipid metabolism	glycolic acid	organic acid
2-monopalmitin	lipid metabolism	hexaric acid	organic acid
dodecanol	lipid metabolism	maleic acid	organic acid
ethanolamine	lipid metabolism	syringic acid	organic acid
glycerol-alpha-phosphate	lipid metabolism	tartaric acid	organic acid
monomyristin	lipid metabolism	cholesterol	sterol
phosphoethanolamine	lipid metabolism	dihydrocholesterol	sterol
phytol	lipid metabolism	squalene	sterol
propane-1,3-diol NIST	lipid metabolism	stigmasterol	sterol
3-aminoisobutyric acid	nucleotide metabolism	beta-gentiobiose	sugar
3-hydroxypropionic acid	nucleotide metabolism	cellobiose	sugar
5,6-dihydrouracil	nucleotide metabolism	fructose	sugar
7-methylguanine NIST	nucleotide metabolism	glucose	sugar
adenine	nucleotide metabolism	glycerol-3-galactoside	sugar
adenosine	nucleotide metabolism	levoglucosan	sugar
adenosine-5-monophosphate	nucleotide metabolism	lyxose	sugar
cytosin	nucleotide metabolism	maltose	sugar
guanosine	nucleotide metabolism	maltotriose	sugar
inosine	nucleotide metabolism	mannose	sugar
orotic acid	nucleotide metabolism	N-acetyl-D-mannosamine	sugar
pseudo uridine	nucleotide metabolism	raffinose	sugar
ribose	nucleotide metabolism	tagatose	sugar
thymidine	nucleotide metabolism	trehalose	sugar
thymine	nucleotide metabolism	xylose	sugar
uracil	nucleotide metabolism	xylulose NIST	sugar
uric acid	nucleotide metabolism	galacturonic acid	sugar acid
uridine	nucleotide metabolism	gluconic acid	sugar acid

gluconic acid lactone	sugar acid
lactobionic acid	sugar acid
ribonic acid	sugar acid
saccharic acid	sugar acid
threonic acid	sugar acid
UDP-glucuronic acid	sugar acid
xylonic acid	sugar acid
erythritol	sugar alcohol
erythrose	sugar alcohol
fucose	sugar alcohol
galactinol	sugar alcohol
hexitol	sugar alcohol
isothreitol	sugar alcohol
lactitol	sugar alcohol
lyxitol	sugar alcohol
mannitol	sugar alcohol
palatinitol	sugar alcohol
xylitol	sugar alcohol
4-pyridoxic acid	vitamin metabolism
delta-tocopherol NIST	vitamin metabolism
hexuronic acid1	vitamin metabolism
nicotinamide	vitamin metabolism
nicotinic acid	vitamin metabolism
tocopherol alpha-	vitamin metabolism
tocopherol beta NIST	vitamin metabolism
tocopherol gamma-	vitamin metabolism
1,2-anhydro-myo-inositol NIST	xother
2-deoxypentitol NIST	xother
2-hydroxypyrazinyl-2-propenoic acid ethyl ester NIST	xother
4-hydroxymandelic acid	xother
5-hydroxymethyl-2-furoic acid NIST	xother
6-hydroxynicotinic acid	xother
acetophenone NIST	xother
allantoic acid	xother
alloxanoic acid NIST	xother
aminomalonate	xother
beta-mannosylglycerate	xother
butyrolactam NIST	xother
caffeine	xother
conduritol-beta-epoxide	xother
creatinine	xother
docosahexaenoic acid	xother
epsilon-caprolactam	xother
hexadecylglycerol NIST	xother
hydroxycarbamate NIST	xother
hydroxylamine	xother
indole-3-acetate	xother
inositol-4-monophosphate	xother
isohexonic acid	xother
isothreonic acid	xother
maleimide	xother
malonic acid	xother
methyl O-D-galactopyranoside	xother
methyltetrahydrophenanthrenone1 NIST	xother
parabanic acid NIST	xother
sebacic acid, di(2-octyl) ester NIST	xother
tyrosol	xother
xylonolactone NIST	xother
zymosterol	xother
